# Water Bottle–Shaped Heart in a Five-Year-Old Boy

**DOI:** 10.4269/ajtmh.18-0956

**Published:** 2019-07

**Authors:** B. Jason Brotherton, Esther W. Kimani-Mangoli, Arianna McLain Shirk

**Affiliations:** 1 Department of Internal Medicine, AIC Kijabe Hospital, Kijabe, Kenya;; 2 Department of Paediatrics, AIC Kijabe Hospital, Kijabe, Kenya;; 3 University of Alabama Birmingham, Birmingham, Alabama

A 5-year-old previously healthy Somali boy presented to our mission hospital in rural Kenya with a 2 week history of a gradual onset of fever, chest discomfort, and difficulty in breathing, accompanied by nonproductive cough, easy fatiguability, and loss of appetite.

Vital signs were as follows: temperature 39.6°C, heart rate 105, respiratory rate 40, oxygen saturation 92% on room air, and weight 13 kg with a mid-upper arm circumference of 11.5 (indicating severe malnutrition). Physical examination was notable for a thin, ill-appearing boy sitting upright in bed. Heart sounds were muffled; peripheral pulses were full. Breath sounds were clear despite being tachypneic. The rest of his physical examination was normal for age.

**Figure 1.**
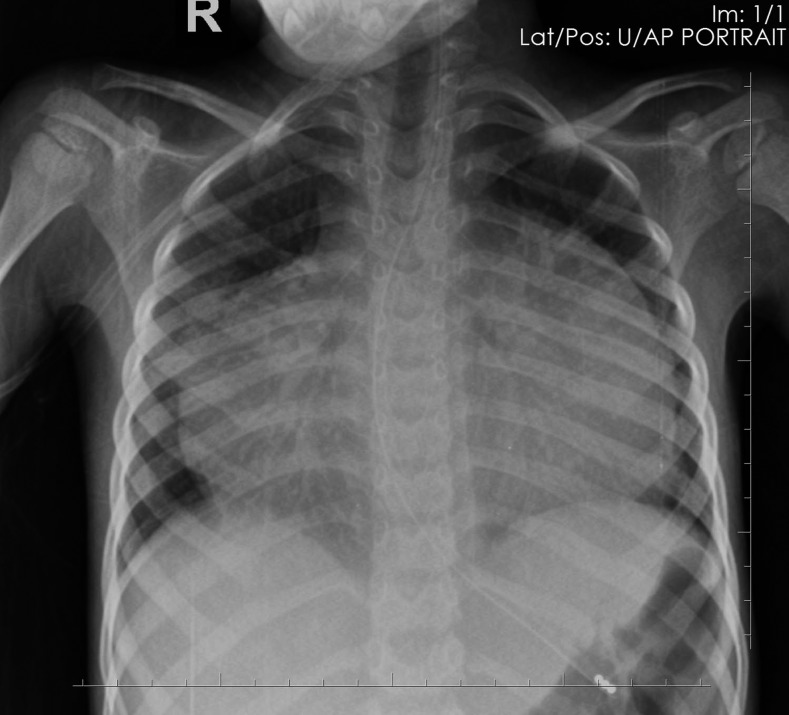
Chest radiograph showing water bottle shaped heart.

**Figure 2.**
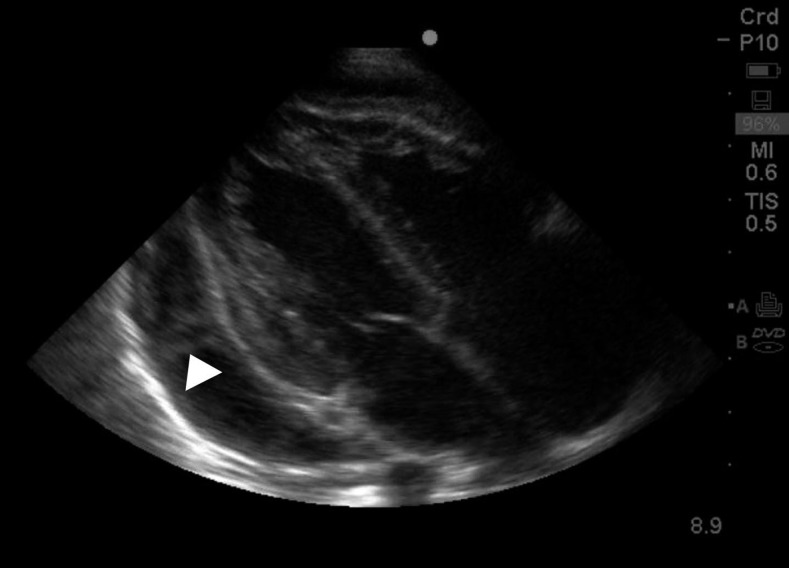
Parasternal long axis view showing moderate pericardial effusion and fibrinous exudate (arrow).

Laboratory investigations were significant for a white blood cell count of 32,300 mm^3^ (75% granulocytes, 14% lymphocytes, and 11% monocytes), hemoglobin 8.2 g/dL, and platelets 393,000/mm^3^. Testing for HIV by rapid antigen test and for tuberculosis (TB) via GeneXpert of a gastric aspirate was negative. Chest radiograph showed an enlarged, globular cardiac silhouette (Figure 1) with unremarkable lung fields. Bedside echocardiography showed good contractility, normal appearing valves, and a moderate pericardial effusion with extensive fibrinous exudate (arrow) extending from the pericardium to the epicardium (Figure 2). A clinical diagnosis of extrapulmonary TB with tuberculous pericarditis was made.

Pericarditis is a well-known extrapulmonary manifestation of TB and the most common cause of pericardial effusion in the resource-limited setting.^1^ Despite this boy having the classic water bottle appearance to the cardiac silhouette, radiography has low sensitivity for detecting pericardial effusion.^2^ Echocardiogram is the definitive test to confirm the presence of pericardial effusion, especially when fibrinous material is observed.

He was started on four drug anti-TB regimen and prednisone 2 mg/kg for the pericardial effusion. A Cochrane review from 2017 suggested that HIV-negative individuals with TB pericarditis may have reduced incidence of death if treated with corticosteroids.^3^

**Figure 3.**
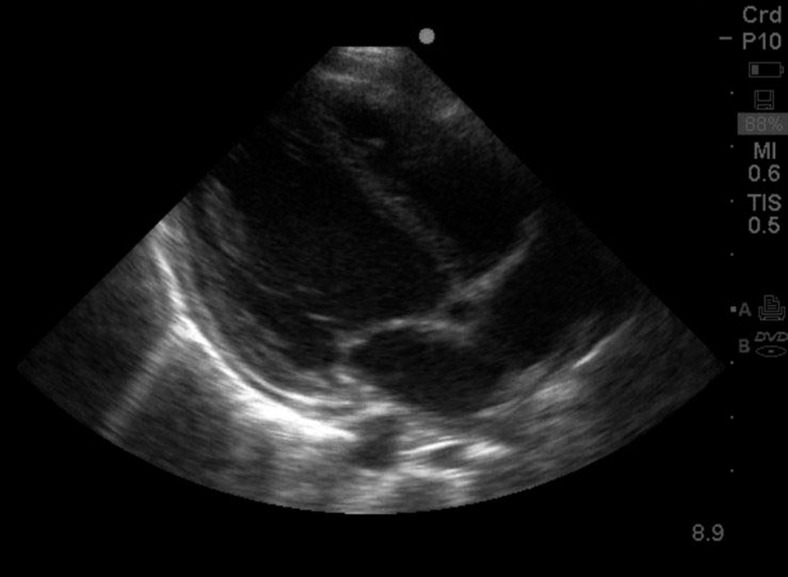
Repeat parasternal long axis view showing resolution of pericardial effusion.

Repeat echocardiogram after 14 days of therapy showed near complete resolution of the effusion (Figure 3). He was discharged on day 22 of hospitalization.
